# Molecular and biological characterization of a novel partitivirus from *Talaromyces pinophilus*

**DOI:** 10.1016/j.virusres.2024.199351

**Published:** 2024-03-11

**Authors:** Sidra Hassan, Urayama Syun-ichi, Saba Shabeer, Tahseen Ali Kiran, Chien-Fu Wu, Hiromitsu Moriyama, Robert H.A. Coutts, Ioly Kotta Loizou, Atif Jamal

**Affiliations:** a Department of Plant and Environmental Protection, PARC Institute of Advanced Studies in Agriculture (Affiliated with Quaid-i-Azam University), National Agricultural Research Centre, Islamabad 45500, Pakistan; b Laboratory of Fungal Interaction and Molecular Biology (donated by IFO), Department of Life and Environmental Sciences, University of Tsukuba, 1-1-1 Tennodai, Tsukuba, Ibaraki 305-8577, Japan; c Department of Bioscience, COMSATS University, Islamabad 44000, Pakistan; d Laboratory of Molecular and Cellular Biology, Department of Applied Biological Sciences, Tokyo University of Agriculture & Technology, 3-5-8, Saiwaicho, Fuchu, Tokyo 184-8509, Japan; e Department of Clinical, Pharmaceutical & Biological Science, School of Life and Medical Sciences, University of Hertfordshire, AL10 9AB, Hatfield, United Kingdom; f Department of Life Sciences, Faculty of Natural Sciences, Imperial College London, SW7 2AZ, London, United Kingdom; g Crop Diseases Research Institute (CDRI), National Agricultural Research Centre, Park Road, Islamabad 45500, Pakistan

**Keywords:** *Talaromyces pinophilus*, Double-stranded RNA, Satellite RNA, *Partitiviridae*, Zetapartitivirus, Growth, Metabolism, Pigmentation

## Abstract

*Talaromyces* spp. have a worldwide distribution, are ecologically diverse and have been isolated from numerous different substrates. *Talaromyces* spp. are considered biotechnologically important due to their ability to produce a range of enzymes and pigments. *Talaromyces pinophilus*, belonging to genus *Talaromyces* and family *Trichocomaceae*, is known for producing several important bioactive metabolites. Here we report the isolation and characterisation of a partitivirus from *T. pinophilus* which we have nominated *Talaromyces pinophilus* partitivirus-1 (TpPV-1). TpPV-1 possesses a genome consisting of three double stranded (ds) RNA segments i.e., dsRNAs1-3, 1824 bp, 1638 bp and 1451 bp respectively, which are encapsidated in icosahedral particles 35 nm in diameter. Both dsRNA1 and dsRNA2 contain a single open reading frame (ORF) encoding respectively a 572 amino acid (aa) protein of 65 kDa and a 504 aa protein of 50 kDa. The third segment (dsRNA3) is potentially a satellite RNA. Phylogenetic analysis revealed that the TpPV-1 belongs to the family *Partitiviridae* in the proposed genus Zetapartitivirus. TpPV-1 infection decreases the mycelial growth rate of the host fungus and alters pigmentation as indicated by time course experiments performed on a range of different solid media comparing virus-infected and virus-free isogenic lines. This is the first report of mycovirus infection in *T. pinophilus* and may provide insights into understanding the effect of the mycovirus on the production of enzymes and pigments by the host fungus.

## Highlights

• Mycovirus infection was for the first time shown in *Talaromyces pinophilus*.
• The mycovirus was tentatively named *Talaromyces pinophilus* partitivirus 1 (TpPV-1).
• TpPV-1 has a tri-segmented genome encapsidated in isometric virions.
• TpPV-1 infection reduces host growth and affects pigment production.

## 1 Introduction

Mycoviruses are distributed widely in fungi and are prevalent in genera including *Alternaria, Aspergillus, Botrytis, Fusarium, Mucor, Penicillium, Rhizopus, Sclerotinia*, and *Trichoderma* (Ghabrial, 1998; Sutela et al., 2019). Mycoviruses have single stranded (ss) RNA or DNA or double stranded (ds) RNA genomes (Yu et al., 2010; Liu et al., 2014) and transmission occurs either vertically *via* spores or horizontally *via* hyphal anastomosis but rarely extracellularly (Yu et al., 2013). Mycoviruses are often latent in their hosts but many have the ability to alter the morphology, growth or pathogenicity of the host fungus and cause hypervirulence or hypovirulence (Ghabrial and Suzuki, 2009; Xie and Jiang, 2014). Mycoviruses may modulate their host transcription profile and these changes, when combined with environmental factors, may produce favourable phenotypic characteristics for us, the host, and in certain situations, for both of us (Kotta-Loizou, 2021). For instance, Cryphonectria hypovirus 1 (CHV-1) causes hypovirulence in *Cryphonectria parasitica* and was used to successfully control the chestnut blight disease in Europe by horizontal transmission to virulent, virus-free strains (Suzuki et al., 2021).

Some viruses, including mycoviruses, have been reported to harbour additional RNA segments termed satellite viruses or satellite RNAs. Satellite viruses and satellite RNAs have little or no sequence homology to the helper virus; the former depend on the helper virus solely for replication and the latter for both replication and encapsidation. Conversely, defective interfering RNAs are derived from the helper virus genome and typically interfere with the helper virus accumulation (Hu et al., 2009; Liu et al., 2015). Although these additional RNA segments are non-essential for helper virus reproduction, they may modulate interactions between the host and the helper virus (Gnanasekaran and Chakraborty, 2018; Wang and Smith, 2016). For instance, satellite dsRNAs associated with mycoviruses in the family *Totiviridae* encode toxins, which are secreted from ‘killer’ yeasts that harbour these dsRNA elements and kill ‘sensitive’ yeasts that do not. The killer yeast system was discovered for the first time in *Saccharomyces cerevisiae*, a yeast of biotechnological importance in baking, brewing and wine-making, offers an advantage to the host in competition for nutrients (Baeza et al., 2008; Crabtree et al., 2023).

The genus *Talaromyces* is currently considered monophyletic but is constantly expanding with increased knowledge of new isolates from all over the world (Sun et al., 2020). Soil is the main habitat for *Talaromyces* spp., but new species have been isolated from other environments including indoor air, dust, clinical samples, plants, seeds, leaf litter, honey, pollen, and stingless bee nests, illustrating the ecological adaptability of this genus (Sun et al., 2020; Chen et al., 2016; Yilmaz et al., 2016; Samson et al., 2011).

The ability of *Talaromyces* spp. to produce enzymes and soluble pigments renders them important for biotechnological purposes. For instance, *T. rugulosus* produces β-rutinosidase and phosphatase (Narikawa et al., 2000; Reyes et al., 1999), *T. pinophilus* produces endoglucanase, cellulase, xylanase and laccase (Li et al., 2017), *T. funiculosus* produces cellulase and *T. cellulolyticus* (*Acremonium cellulolyticus*) is an important cellulose-degrading fungus used for biomass degradation (de Benito et al., 2017; Houbraken et al., 2014). *T. pinophilus*, is also used in the production of bioactive metabolites including alkaloids, esters, furanosteroids lactones, polyketides, terpenoids, and tetraenes (Li et al., 2017; Vinale et al., 2017; Zhai et al., 2016). Recently, new metabolites were reported including alkanes, amides, aromatic compounds, fatty acids, furans, ketones, acetic acid, 9-octadecenamide, hydrazine, hexadecane, nonadecane, eicosane and undecanoic acid methyl ester, with antimicrobial, antioxidant and anti-inflammatory properties and widely used as plant growth promoters and as biofuel (Adelusi et al., 2022).

Natural fungal pigments are currently receiving attention mainly due to their wide range of applications in different industries. In addition to their colouring properties, other beneficial attributes include antimicrobial, anticancer, antioxidant, and cytotoxic activity, expanding the use of myco-pigments in different sectors (Lagashetti et al., 2019). In particular, *T. pinophilus* together with other *Talaromyces* spp. produce *Monascus*-like azaphilone pigments, a mixture of secondary metabolites used as a food colorant (Caro et al., 2016). Additionally, *T. pinophilus* reduces the absorption of heavy metals by wheat from soil amended with sewage sludge and boosts photosynthetic pigment production and plant growth (El-Shahir et al., 2021).

*Talaromyces* spp. have only been reported to harbour a small number of mycoviruses to date. The presence of a mycovirus in *Talaromyces* spp. was first reported in 2018, when *T. marneffei* was found to be infected with Talaromyces marneffei partitivirus-1 (TmPV-1) causing hypervirulence (Lau et al., 2018). *T. amestolkiae*, ubiquitous in soil, plants, air and food (Yilmaz et al., 2012), was found to be infected with the six-segmented Talaromyces amestolkiae polymycovirus 1 (TaPmV-1) (Teng et al., 2022). No other mycoviruses have been reported in *Talaromyces* spp.

In the present study we report the first mycovirus infection of *T. pinophilus* and explore the role of the mycovirus on the host fungus radial growth, biomass and pigment production.

## 2 Materials and methods

### 2.1 Isolation and identification of fungal isolates

*T. pinophilus* was identified from tobacco soil samples, collected from different regions of Khyber Pakhtunkhwa, Pakistan. A total of 35 soil samples were serially diluted and inoculated on potato dextrose agar (PDA, Millipore) plates supplemented with streptomycin (0.12 g/L) followed by incubation at 28 °C for 5–7 days. Fungal colonies were observed and single inocula from their actively growing regions were transferred for purification to 60 mm PDA plates using inoculation loops and incubated at 28 °C for 3–5 days. The pure cultures that grew on each plate were further used for total nucleic acid extraction (Coenen et al., 1997; Hirai et al., 2021) after microscopic examination for morphological characterization. Fungal mycelia were stored in 40 % (v/v) glycerol at −20 °C for future use. For molecular identification of fungi, the internal transcribed spacer (ITS) regions of nuclear DNA were amplified by polymerase chain reaction (PCR) using ITS1 and ITS4 primers (Vancov and Keen, 2009; White et al., 1990).

### 2.2 Fragmented and primer-ligated dsRNA sequencing (FLDS)

A novel method FLDS was used for the viral genome sequencing as described (Hirai et al., 2021; Urayama et al., 2016).This method consists of physical fragmentation of dsRNA following cellulose column chromatography, synthesis of cDNA by reverse transcription (RT) using a modified RACE method, library construction and amplification of cDNA *via* PCR. Finally, dsRNA sequencing was done using the KAPA Hyper Prep Kit Illumina Platform (Kapa Biosystems, Woburn MA, USA). Illumina NovaSeq 6000/PE150 was used to determine 150 bp of the paired end sequence of each fragment. Raw sequencing data were processed through the FLDS pipeline (available in GitHub, https://github.com/takakiy/FLDS). CLC Genomic Workbench version 11.0 was used for de novo assembly of contigs >300 nt (other options were default). Full-length recovery of dsRNA segments was confirmed by manual analysis using CLC Genomic Workbench version 11.0, Genetyx version 14, and Tablet viewer version 1.19.09.03.

### 2.3 Multiple sequence alignment, phylogenetic analysis, and secondary structure prediction

The online Multiple Alignment using Fast Fourier Transform (MAFFT v7.511) tool was used for multiple sequence analysis (Katoh et al., 2019). The Neighbour Joining (NJ) method with a bootstrap value of 1000 replication as implemented by the Molecular Evolutionary Genetics Analysis (MEGA) 11 software was used for phylogenetic analysis. (Tamura et al., 2021). The secondary structures for the 5′ and 3′ termini of TpPV-1 dsRNAs were determined using the online RNAfold (2.5.1) tool (Gruber et al., 2008).

### 2.4 Purification of virus-like particles (VLPs)

VLPs were purified as described previously (Jamal et al., 2019) with some modifications. Approximately 80 g of fungal mycelia was homogenized in 2 volumes of TE buffer (50 mM Tris-Cl pH 7.5, 1 mM EDTA pH 8.0) for 3 min. After filtering, the homogenate was centrifuged at 10,000 × *g* for 20 min. The supernatant was precipitated with 10 % (w/v) PEG-6000 and 0.6 M NaCl overnight at 4 °C followed by centrifugation at 10,000 × *g* for 20 min. The pellet containing the virus was resuspended in TE buffer and re-centrifuged for 20 min at 10,0000 × *g* and the supernatant containing the virus was subjected to ultracentrifugation at 105,000 × *g* for 90 min. TE buffer (1 mL) was used for the re-suspension of purified pelleted virus and transferred to 1.5 mL Eppendorf tubes. The tubes were again centrifuged at 10,000 × *g* for 20 min to further clarify the supernatant containing the virus. Viral dsRNA was extracted with phenol-chloroform-isoamyl alcohol, electrophoresed on 1 % (w/v) agarose-stained gel, and visualized under ultraviolet (UV) light.

### 2.5 Transmission electron microscopy (TEM) and sodium dodecyl sulphate-polyacrylamide electrophoresis (SDS-PAGE)

TEM operating at 120 kV using a LAB6 filament was performed for the visualization of purified VLPs on carbon Film 300 Mesh copper grids. SDS-PAGE was performed with purified VLPs (Shamsi et al., 2019) followed by silver staining.

### 2.6 Generating virus-infected and virus-free isogenic lines

Attempts were made to cure the fungus from mycoviral infection. Methods such as heat shock and hyphal tipping (Tran et al., 2019) were used to obtain a virus-cured isogenic line but none of the attempts were successful. Therefore, a paired culture technique was utilized by inoculating side by side on a PDA plate the T40 virus-infected (donor) and the T140 virus-free (recipient) strains, both sourced from tobacco soil samples, and allowing the cultures to grow and fuse (Wu et al., 2017). Cultures were incubated at 28 °C for 5–7 days and three different regions were selected from the recipient and donor sides on days 3, 5, 6 and 7 followed by sub-culturing on fresh PDA plates. The cultures were subsequently grown in potato dextrose broth (PDB), followed by total nucleic extraction as above.

### 2.7 Radial expansion, biomass production and metabolic activity assays

Spore suspensions from the T140 virus-free and virus-infected isogenic lines were quantified using a hemocytometer. Equal numbers of spores were used for radial expansion, biomass production and metabolic activity assays.

Radial growth expansion assays were performed to investigate the effect of mycovirus infection on *T. pinophilus* morphology and growth (Kim et al., 2015; Song et al., 2016). Several different media were investigated including Czapek–Dox agar complete (CDA-CM) and minimal (CDA-MM), PDA, Sabouraud dextrose agar (SDA) and yeast extract and sucrose (YES) agar. In each case, 10 μL of equal numbers of spores (500 spores/μL) of both virus-infected and virus-free isogenic lines were centrally inoculated and incubated at 28 °C in the dark. Colony morphology was observed, and radii were measured every 24 h over a period of 6 days.

For biomass production assays, 50 μL of equal numbers of spores (500 spores/μL) of virus-infected and virus-free isogenic lines were inoculated into 10 mL of PDB in 50 mL Falcon tubes and incubated at 28 °C in the dark. Once visible mycelial mats had grown for 2 weeks post inoculation, the cultures were filtered through Miracloth, and wet mycelial weights were measured. Mycelia were then wrapped in filter paper and dried for 24 h before dry mycelial weights were measured.

A 2,3-bis(2-methoxy-4-nitro-5-sulfophenyl)-5-carboxanilide-2H-tetrazolium (XTT) metabolic assay was performed as described previously (Scudiero et al., 1988; Ferreira et al., 2015) to compare the metabolic activity of virus-infected and virus-free isogenic lines. Equal numbers of spores (10^7^) were seeded in 96-well plates containing YES, malt extract broth (MEB) or Czapek–Dox minimal media (CD-MM) and allowed to grow at 28 °C for 24 h. XTT-menadione-liquid media solution was added to each well and the 96-well plates were incubated in the dark for 1 h. The supernatants from each well were transferred to fresh 96-well and absorbance measured using a plate reader at 490 nm.

All experiments were performed in triplicate and results were analysed for statistical significance using Student's *t*-test or two-way analysis of variance (ANOVA) in GraphPad Prism.

## 3 Results

### 3.1 Identification of a dsRNA mycovirus from a soil isolate of *T. pinophilus*

**Fig. 1:**
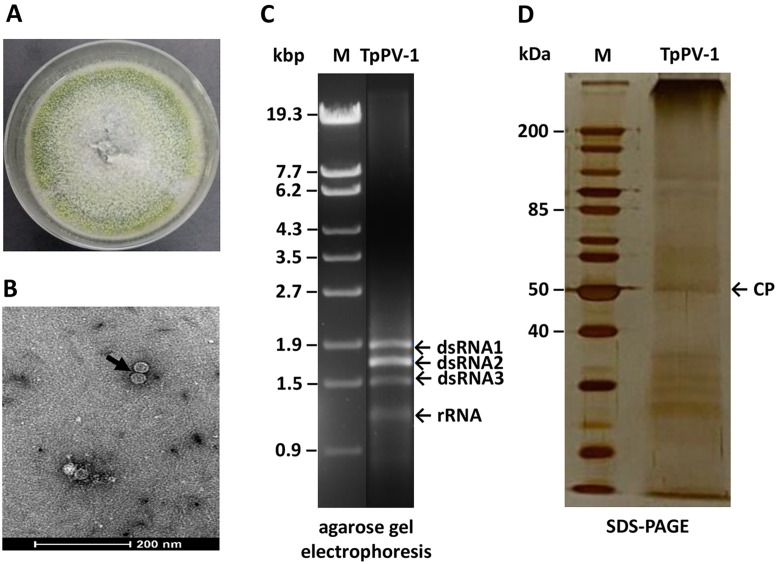
TpPV-1 components. (A) Colony morphology of *T. pinophilus* isolate T40 on PDA. (B) Icosahedral VLPs from *T. pinophilus* with a diameter of 35 nm as shown by TEM. (C) Electrophoretic profile of TpPV-1 dsRNA elements isolated from *T. pinophilus* on a 1 % (w/v) agarose gel, reveals three dsRNA bands ca. 1.9, 1.7 and 1.5 kbp in size. The molecular sizes of λ-EcoT141-digested DNA marker are indicated to the left of the gel. (D) Electrophoretic profile of TpPV-1 coat protein on a 10 % (^w^/_v_) SDS-PAGE which is 50 kDa in size. The molecular sizes of PAGE Ruler unstained protein ladder are indicated to the left of the gel.

A total of 35 tobacco field soil samples were collected, resulting in 238 fungal isolates representative of genera including *Rhizopus, Acrophialophora, Aspergillus, Fusarium, Morteirella, Penicillium, Talaromyces*, and *Trichoderma* species. Following screening for mycovirus infection, 3 isolates tested positive and were found to contain the genomic RNA of dsRNA viruses or replicative form dsRNA of ssRNA viruses. In particular, *T. pinophilus* isolate T40 was found to harbour dsRNA elements between 1 and 2 kb in size (Fig. S1). The colony morphology of the virus-infected *T. pinophilus* isolate and its dsRNA electrophoretic profile are shown in Fig. 1A and C, respectively. TEM visualisation revealed isometric VLPs 35 nm in diameter (Fig. 1B). The viral protein component was subjected to SDS-PAGE and an expected band *ca*. 50 kDa in size was observed following silver staining (Fig. 1D).

### 3.2 Molecular characterisation of a novel partitivirus infecting *T. pinophilus*

The purified dsRNA elements were subjected to FLDS for complete sequence determination. The complete individual sequences of three dsRNAs, 1824, 1638 and 1452 bp in size, similar to dsRNA viruses in the family *Partitiviridae*, was identified. The smallest dsRNA segment, which might be satellite RNA, is not present in all partitiviruses. The virus was nominated *Talaromyces pinophilus* partitivirus-1 (TpPV-1) and the complete nucleotide sequences of dsRNAs 1–3 were submitted to DNA Data Bank of Japan (DDBJ) with accession numbers LC768111, LC768112 and LC768113 respectively.

**Fig. 2:**
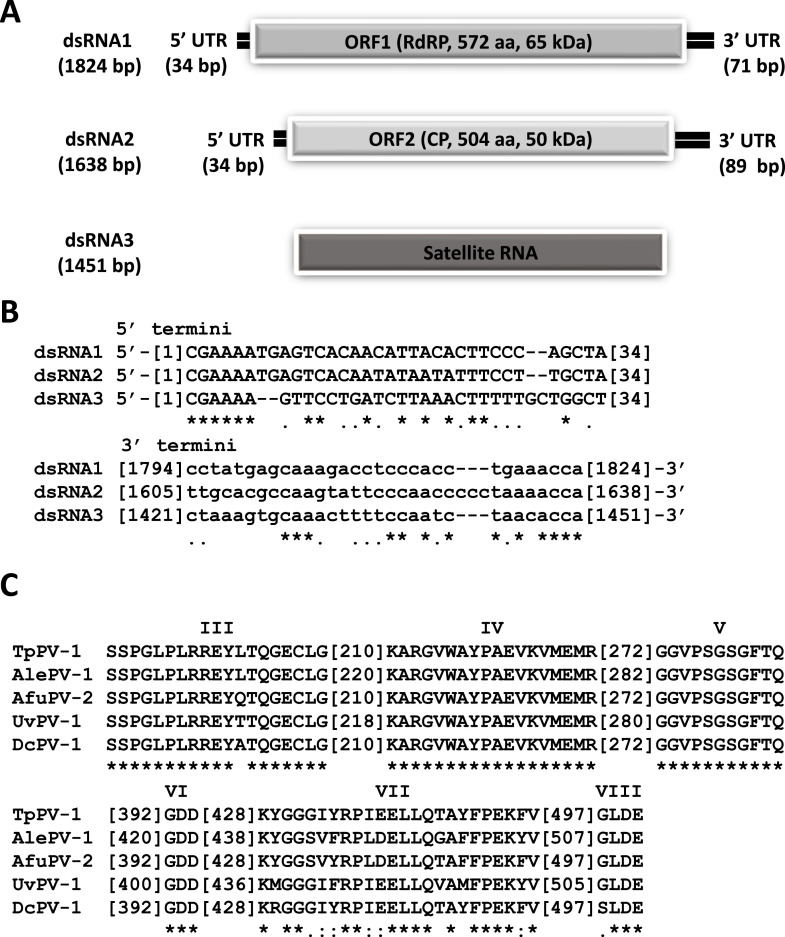
TpPV-1 genomic organisation. (A) Schematic representation of TpPV-1 dsRNA 1, dsRNA 2 and dsRNA3. Genomic dsRNA1 and dsRNA2 are shown as a black line and each ORF as a grey box. Satellite dsRNA is shown as a dark grey box. (B) Nucleotide sequence alignment of the 5′- and 3′-termini of the TpPV-1 dsRNAs 1, 2 and 3 using MAFFT. Asterisks indicate identical nucleotides; single dots indicate purines or pyrimidines. (C) Amino acid sequence alignment of the core RdRP motifs (III-VIII) of TpPV-1 and selected members in the family *Partitiviridae* using MAFFT. Asterisks indicate identical residues; colons indicate highly conserved residues and single dots indicate less conserved but related residues.

Sequence analysis revealed that both dsRNA1 and dsRNA2 have single ORFs encoding proteins of 572 aa (65 kDa) and 504 aa (50 kDa) respectively (Fig. 2A). The Basic Local Alignment Sequence Tool for proteins (BLASTp) was used to search public databases and revealed that the TpPV-1 dsRNA 1 ORF encodes a protein that shares clear similarities with the RNA-dependent RNA polymerases (RdRP) of several other viruses in the family *Partitiviridae*, including Botryosphaeria dothidea virus 1 (AGZ84316; 73.95 % sequence identity), Aspergillus lentulus partitivirus 1 (BCH36641; 72.55 % sequence identity), Alternaria alternata partitivirus 2 (APT70073; 72.20 % sequence identity), Delitschia confertaspora partitivirus 1 (AZT88584; 72.20 % sequence identity) and Aspergillus fumigatus partitivirus 2 (AXE72935; 71.55 % sequence identity) (Table S1). Multiple alignment of the putative TpPV-1 RdRP sequence and other related sequences from the family *Partitiviridae* was performed and revealed the presence of six conserved RdRP motifs (III-VIII) (Bruenn, 1993) (Fig. 2C). Similarities between the putative protein encoded by the TpPV-1 dsRNA 2 ORF and other *Partitiviridae* coat proteins (CP) were found in Botryosphaeria dothidea virus 1 (AGZ84317; 61.10 % sequence identity), Aspergillus lentulus partitivirus 1 (BCH36642; 57.05 % sequence identity), Alternaria alternata partitivirus 2 (APT70074; 53.79 % sequence identity), Delitschia confertaspora partitivirus 1 (AZT88585; 53.78 % sequence identity), and Aspergillus fumigatus partitivirus 2 (UJQ88259; 52.46 % sequence identity) (Table S1).

**Fig. 3:**
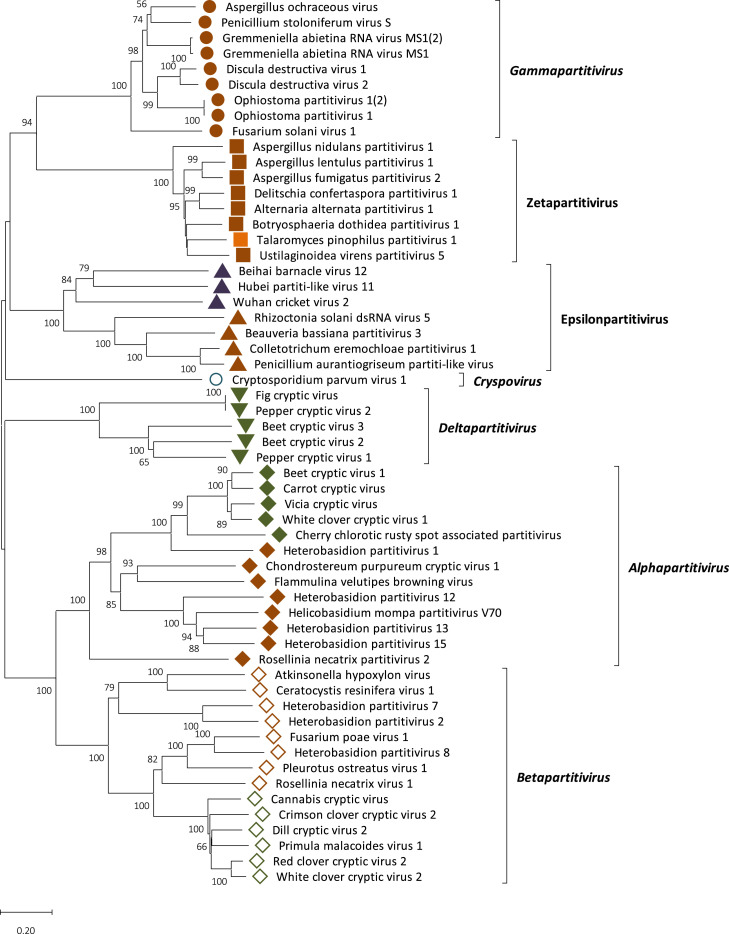
TpPV-1 phylogeny. Phylogenetic analysis of TpPV-1 and selected members of the family *Partitiviridae* based on their RdRP amino acid sequences. Using MUSCLE, as implemented by MEGA 11, a multiple alignment of RdRP amino acid sequences was created. MEGA 11 was used to create a neighbour joining (NJ) phylogenetic tree. Bootstrap percentages (1000 replications) above 50 % are displayed. Tips labelled with brown, green, purple and blue shapes indicate that the virus host is respectively fungal, plant, insect or protozoan. The orange square indicates the position of TpPV-1.

The phylogenetic position of TpPV-1 was examined by constructing a phylogenetic tree using RdRP sequences of members in the family *Partitiviridae* (Fig. 3). The tree shows that TpPV-1 and other related viruses (Aspergillus lentulus partitivirus 1, Botryosphaeria dothidea virus 1, Delitschia confertaspora partitivirus 1, Aspergillus fumigatus partitivirus 2 and Alternaria alternata partitivirus 2) (Table S1) cluster in an as yet unclassified group termed Zetapartitivirus outside the five known genera of *Partitiviridae* (Jiang et al., 2019).

The 5′-untranslated regions (UTRs) of TpPV-1 dsRNA 1 and 2 are 34 bp long and show conserved nucleotides at the 5′ terminus (CGAAAATGAGTCACAA) (Fig. 2B). The 3′-UTRs differ in length, 71 bp for dsRNA 1 and 89 bp for dsRNA 2, and contain conserved nucleotide stretches (AAACCA) but otherwise there is little sequence conservation (Fig. 2B). This is consistent with the findings for multi-component RNA viruses, which show that the viral RdRP needs to recognise the terminal sequences at the 5′-termini ends for viral RNA recognition and replication (Nibert et al., 2014). Possible secondary structures were predicted, and a stem loop structure was observed for both the 5′ and 3′ termini of dsRNA 1 and 2 (Fig. S2). RNA transcripts use complex base pairing patterns to fold into secondary structures. These secondary structures form an essential node of biological regulation by giving a variety of RNAs scaffolding, ligand binding, and catalytic properties.These structures are additionally known to provide the genome with stability and may be involved in viral replication and assembly (Vandivier et al., 2016) .

### 3.3 Horizontal transfer of TpPV-1 to a virus-free *T. pinophilus* isolate

**Fig. 4:**
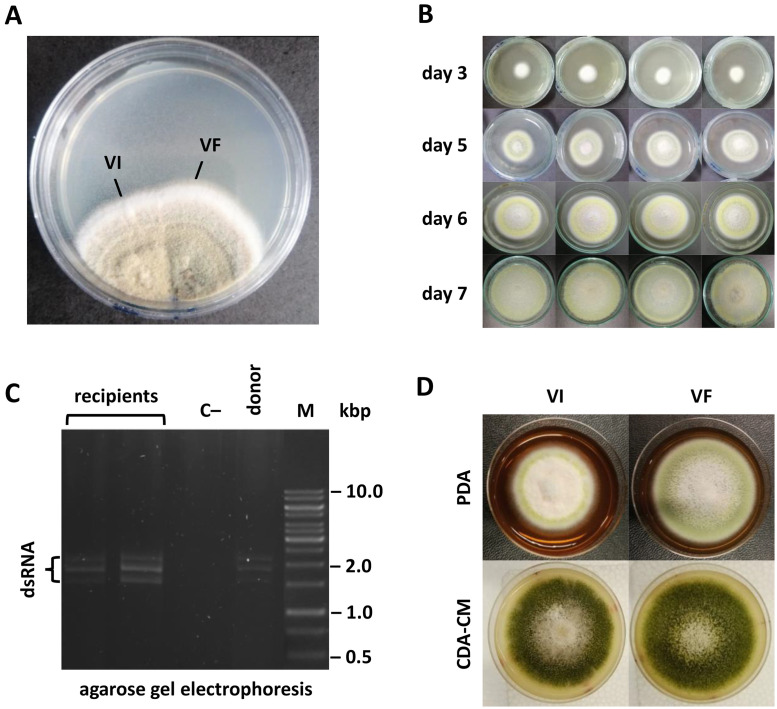
TpPV-1 horizontal transfer. (A) Fusion of two isolates of *Talaromyces* to transfer TpPV-1 from the T40, virus-infected isolate to the T140, virus-free isolate. (B) Morphology of the colonies picked from the recipient zone grown on PDA at different times post inoculation. (C) Gel electrophoretic profile confirming successful transfer of TpPV-1 from donor to recipient *via* hyphal anastomosis. (D) Colony morphologies of virus-infected and virus-free isolates grown on PDA and CDA-CM showing variation in mycelial growth.

Single spore isolation and heat treatment failed to cure TpPV-1, so hyphal fusion was utilized to transfer TpPV-1 from the virus-infected isolate T40 to a virus-free isolate T140. The latter was chosen based on comparable morphology and the 99.8 % similarity in its ITS sequence between T40 (ON024678) and T140 (ON247408). TpPV-1 was successively transferred horizontally by hyphal fusion from the virus-infected isolate T40 to the virus-free isolate T140 (Fig. 4B) which was confirmed following a comparison of the total nucleic acid electrophoretic profile of the two isolates (Fig. 4C). The morphologies of both virus-infected and virus-free T140 isolate are shown in Fig. 4D.

### 3.4 Effects of TpPV-1 infection on *T. pinophilus* radial growth, biomass, metabolism and pigment synthesis

**Fig. 5:**
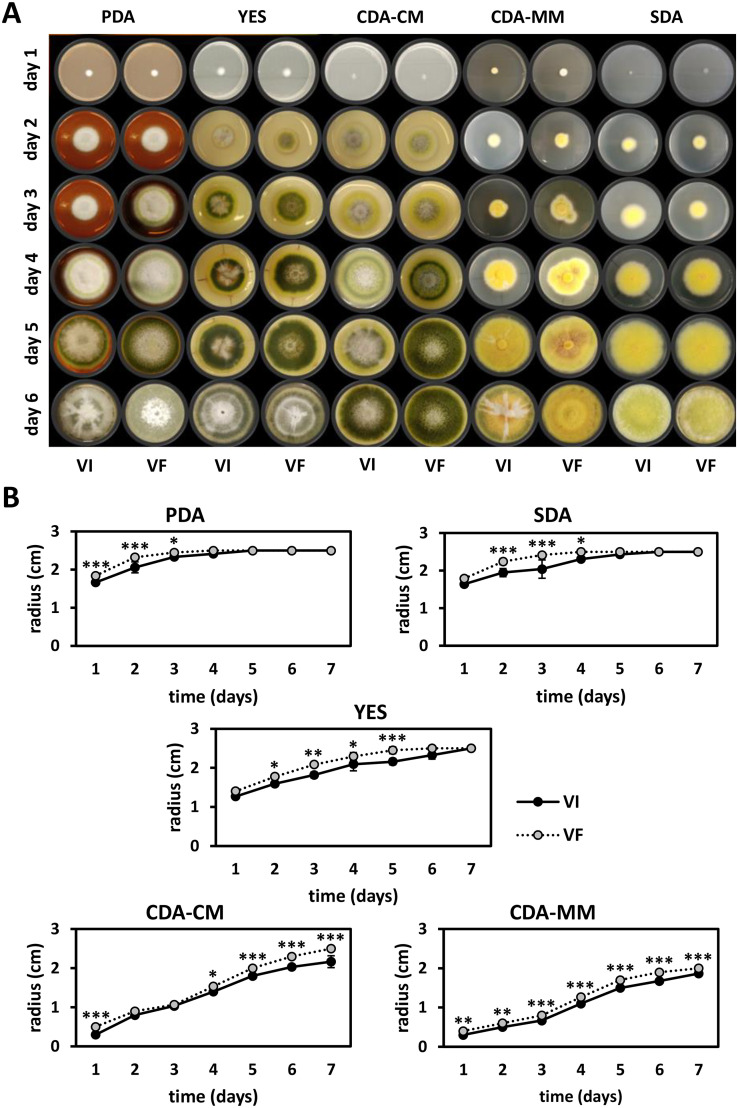
Radial growth of *T. pinophilus* isogenic lines. (A) Colony morphologies and (B) radial growth of *T. pinophilus* virus-infected (VI) and virus-free (VF) isogenic lines on different media, including PDA, SDA, YES, CDA-CM and CDA-MM over a period of 7 days. Asterisks indicate statistical significance following 2-way ANOVA: * indicates *P*-value <0.05; ** indicates *P*-value <0.01; *** indicates *P*-value <0.001.

The radial growth of virus-free (VF) and virus-infected (VI) isogenic lines was measured over a 7-day period on different media. In all cases, VI growth was slower than VF and was confirmed as statistically significant when measured at different time points (2-way ANOVA, *P*-value <0.05). The colony morphologies of the isogenic lines grown on different media are shown together with the graphical representation of the diameters recorded (Fig. 5A and B respectively). There are notable differences in pigmentation between the isogenic lines, dependent on the growth medium (Fig. 5A).

**Fig. 6:**
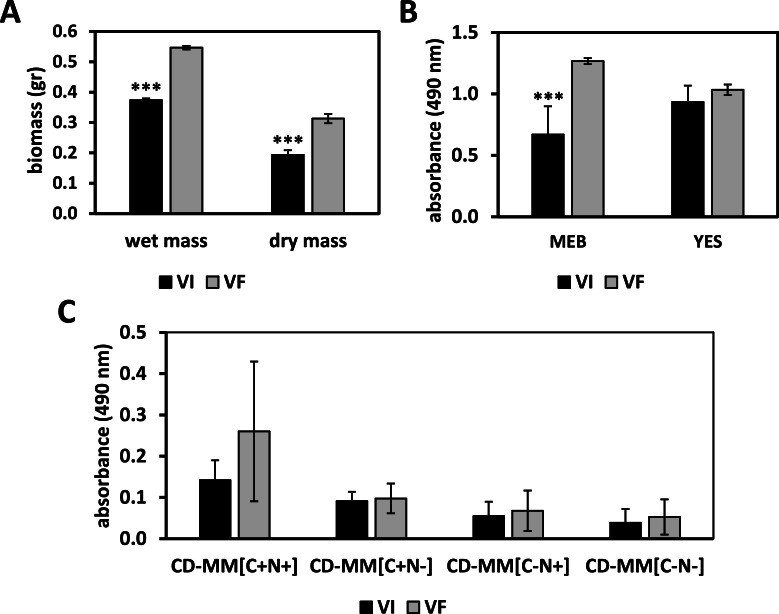
Biomass and metabolism of *T. pinophilus* isogenic lines. (A) Biomass production of *T. pinophilus* virus-infected (VI) and virus-free (VF) isogenic lines in PBD. Asterisks indicate statistical significance following Student's *t* test: *** indicates *P*-value <0.001. (B) Metabolic activity of *T. pinophilus* isogenic lines in MEB and YES. (C) Metabolic activity of *T. pinophilus* isogenic lines in CD-MM with and without carbon and nitrogen sources. Asterisks indicate statistical significance following 2-way ANOVA: *** indicates *P*-value <0.001.

Both wet and dry biomass production was significantly decreased (Student's *t*-test, *P*-value <0.001) in VI as compared to VF in PDB (Fig. 6A). In metabolic assays, the XTT substrate is reduced by a dehydrogenase enzyme produced by metabolically active fungi (Singh et al., 2011) generating a formazan pigment. Metabolic activity was significantly decreased (2-way ANOVA, *P*-value <0.001) in VI as compared to VF in MEB (Fig. 6B), while no statistically significant differences in the metabolic activity of the isogenic lines were noted in YES (Fig. 6B) and CD-MM with different formulations (Fig. 6C).

**Fig. 7:**
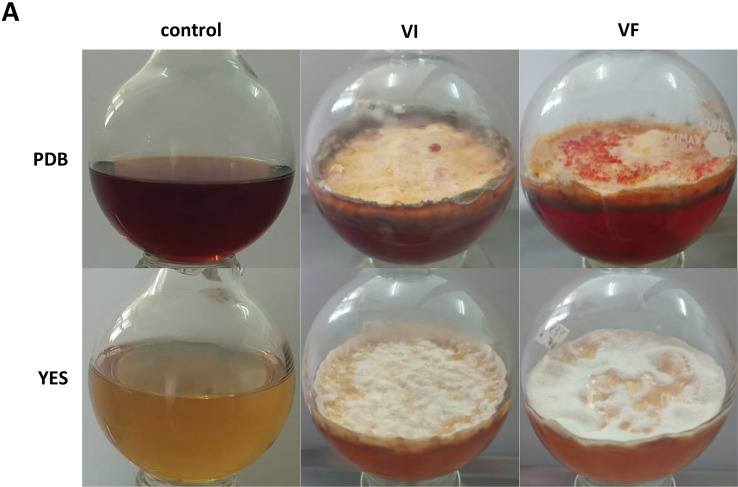
Pigment production by *T. pinophilus* isogenic lines. Virus infected and virus free cultures of *T. pinophilus* in Potato dextrose broth (PDB) and Yeast and sucrose (YES) media, showing colored pigment along with control.

Notably, VI cultures of *T. pinophilus* grown in CD-MM containing sucrose and sodium nitrate as carbon and nitrogen sources respectively were lightly pigmented while cultures deprived of carbon and nitrogen were lighter in color with minimum optical density values recorded by spectrometry presumably because of limited fungal growth. Conversely, VF cultures were darkly pigmented. Substantial differences in pigmentation were found in PDB, where VF produced more orange-red pigments as compared to VI (Fig. 7). These observations suggest a role for TpPV-1 in host fungus metabolism related to pigment production.

## 4 Discussion

*Talaromyces* spp. are ubiquitous worldwide and can be isolated from soil and numerous other sources including indoor air, dust, litter, plants, seeds, pollen, and stingless bee nests (Sun et al., 2022). In this investigation we report the first documentation and characterisation of a novel mycovirus from *T. pinophilus* which comprises three genomic dsRNA segments and is associated with the presence of 35 nm isometric VLPs. We have nominated the virus Talaromyces pinophilus partitivirus-1 (TpPV-1).

Phylogenetic analysis revealed that TpPV-1 is a new member in the family *Partitiviridae*. Construction of phylogenetic trees for RdRP proteins TpPV-1 together with closely related partitiviruses (Fig. 3) indicates that all these viruses cluster in a separate group outside the established genera of *Partitiviridae* and may belong to the proposed genus termed Zetapartitivirus (Gilbert et al., 2019).

Substantial sequence identity of the 5′-UTRs of two genomic dsRNAs, possibly involved in virion packaging, is a characteristic feature of partitiviruses and the same is true for TpPV-1 (Fig. 2B) (Teng et al., 2022; Nibert et al., 2014). The viruses in the family *Partitiviridae* have been reported to be icosahedral in shape and are non-enveloped (45). The RdRp and CP encoded by TpPV-1 dsRNAs 1 and 2 respectively show significant similarity to similar, hypothetical proteins encoded by a group of partitiviruses listed in Table S1. TpPV-1 dsRNA3 represents a satellite RNA, the occurrence of which has been reported before for partitiviruses. Several partitiviruses have been reported to contain satellite viruses or RNA segments including Aspergillus flavus partitivirus 1 (AfPV-1; 3), Aspergillus fumigatus partitivirus-2 (Briddon et al., 2012), Atkinsonella hypoxylon virus (AhV-Seg3*), Discula destructiva virus (DdV-Seg3 and DdV-Seg4), Gremmeniella abietina virus MS1 (GaVMS1-Seg3), Penicillium stoloniferum virus (PsV-F).

Limited information is available concerning the occurrence and characterisation of mycoviruses in *Talaromyces* spp. apart from *T. amestolkiae*, which hosts Talaromyces amestolkiae polymycovirus-1 (TaPmV-1; 22), Talaromyces marneffei partitivirus-1 (TmPV-1; 20) and TpPV-1 investigated here. All three aforementioned mycoviruses altered host virulence, gene expression and pigment production. *Talaromyces* spp. produce structurally related polyketide-based yellow, orange and red pigments (de Oliveira et al., 2021). TaPmV-1 reduced red pigment production by downregulating gene expression (Teng et al., 2022) while TmPV-1 increased host virulence and caused abnormal gene expression (Lau et al., 2018). A comparison between TpPV-1-infected and -free isogenic lines revealed that TpPV-1 altered morphology in culture, decreased host growth and biomass production, and influenced pigmentation, caused by altered gene expression which requires further investigation.

## 5 Conclusion

In summary, this study provides the first report of viral infection in *T. pinophilus*. The molecular characterization indicated that the virus belongs to the proposed genus Zetapartitivirus and has the characteristics of a member of the family *Partitiviridae*. Radial growth, biomass production and XTT metabolic assays indicated that the TpPV-1 reduces the growth of the host and affects pigment production. Further studies are required to understand the role of TpPV-1 in host physiology.

## CRediT authorship contribution statement

**Sidra Hassan:** Writing – original draft, Visualization, Validation, Methodology, Investigation, Funding acquisition, Formal analysis, Data curation. **Urayama Syun-ichi:** Writing – review & editing, Validation, Software, Resources, Methodology, Investigation, Formal analysis, Data curation. **Saba Shabeer:** Writing – review & editing, Validation, Investigation. **Tahseen Ali Kiran:** Writing – review & editing, Validation, Investigation. **Chien-Fu Wu:** Writing – review & editing, Visualization, Validation, Investigation. **Hiromitsu Moriyama:** Writing – review & editing, Supervision. **Robert H.A. Coutts:** Writing – review & editing, Supervision. **Ioly Kotta Loizou:** Writing – review & editing, Validation, Supervision, Resources, Project administration, Funding acquisition, Formal analysis, Data curation. **Atif Jamal:** Writing – review & editing, Supervision, Resources, Project administration, Funding acquisition, Conceptualization.

## Declaration of competing interest

The authors declare that they have no known competing financial interests or personal relationships that could have appeared to influence the work reported in this paper.

## Data Availability

The complete genome of TpPV-1 (dsRNAs 1–3) was submitted to DNA Data Bank of Japan (DDBJ) with accession numbers LC768111, LC768112 and LC768113 respectively.

## Appendix Supplementary materials

Image, application 1
