# Supplementary material for: Molecular and biological characterization of a novel partitivirus from Talaromyces pinophilus
Source: Virus Res. 2024 Mar 11;343:199351. doi: 10.1016/j.virusres.2024.199351 (PMC10982079; doi:10.1016/j.virusres.2024.199351)
Supplement: Supplementary file 1 [file mmc1.pdf]

Table S1. TpPV-1 encoded proteins and their homologues as revealed by BLASTp.

	**dsRNA1 RdRP**			**dsRNA2 CP**		
**Virus**	**Identity (%)**	**E-value**	**Accession**	**Identity (%)**	**E-value**	**Accession**
Botryosphaeria dothidea virus 1						
	73.95	0.0	AGZ84316	61.10	9e-175	AGZ84317
Aspergillus lentulus partitivirus 1						
	72.55	0.0	BCH36641	57.05	0.0	BCH36642
Delitschia confertaspora partitivirus 1						
	72.20	0.0	AZT88584	53.78	3e-164	AZT88585
Aspergillus fumigatus partitivirus 2						
	71.55	0.0	AXE72935	52.46	6e-150	UJQ88259
Alternaria alternata partitivirus 1						
	72.20	0.0	APT70073	53.79	3e-164	APT70074

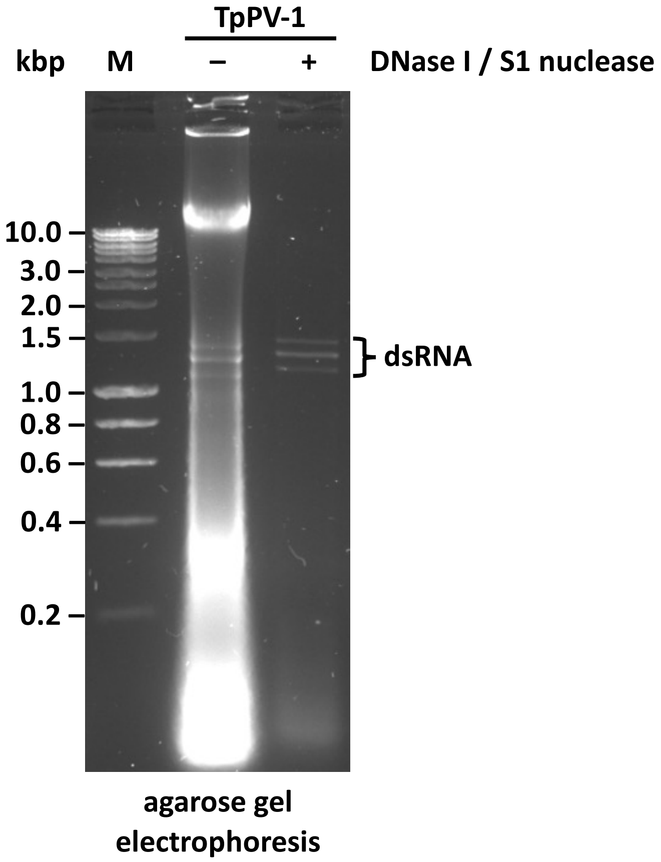

Figure S1. Electrophoretic profile of nucleic acids extracted from *T. pinophilus* on a 1% (w/v) agarose gel before and after treatment with DNase I and S1 nuclease. The molecular sizes of HyperLadder 1kb (Bioline) DNA marker are indicated to the left of the gel.

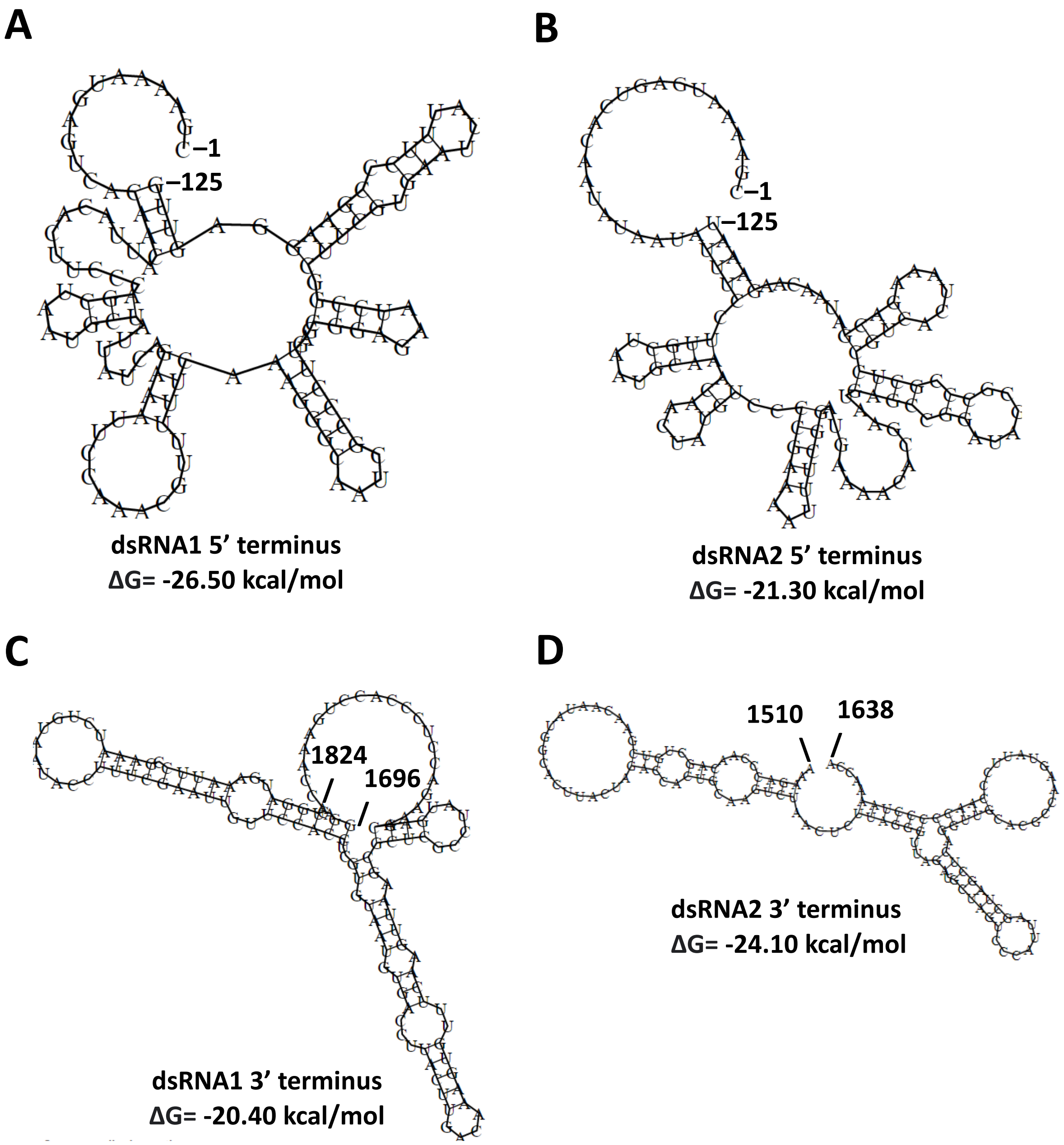

Figure S2. Predicted secondary structures of the 5′ and 3′ UTRs of TpPV-1 dsRNA1 and dsRNA2 using online RNAfold 2.5.1. The minimum free energy (ΔG) is shown with each predicted structure.
